# Does the Brain Care Which Direction We Read? A Cross‐Cultural tDCS Study on Functional Lateralization of Number Processing

**DOI:** 10.1002/brb3.70353

**Published:** 2025-03-09

**Authors:** Narjes Bahreini, Christina Artemenko, Christian Plewnia, Reza Rostami, Hans‐Christoph Nuerk

**Affiliations:** ^1^ Department of Psychology University of Tuebingen Tuebingen Germany; ^2^ German Center for Mental Health (DZPG) Tuebingen Germany; ^3^ Department of Psychiatry and Psychotherapy, Neurophysiology & Interventional Neuropsychiatry University Hospital of Tuebingen Tuebingen Germany; ^4^ Department of Psychology University of Tehran Tehran Iran

**Keywords:** culture, functional lateralization, number processing, reading direction, tDCS

## Abstract

**Purpose:**

The potential influence of culture on functional lateralization was rarely investigated, yet it may be an important factor in our understanding of the human brain. In numerical processing, evidence was found for differential directional preferences of space–number associations in cultures with opposite reading direction systems. This may affect finger‐counting preferences like the starting hand, which in turn have previously been associated with differing lateralization. Such studies raise the question of whether number culture may also play a distinct role in the lateralization of the intraparietal sulcus (IPS), the hallmark region of numerical magnitude processing.

**Method:**

In our preregistered cross‐cultural study, we applied anodal transcranial direct current stimulation (tDCS) over the left versus right IPS to investigate the effect of stimulation as compared to sham in Iranians (with right‐to‐left reading system) and Germans (with left‐to‐right reading system).

**Finding:**

Results indicated no overall effect of stimulation; however, exploratory analyses revealed that tDCS over the left and right IPS facilitated number processing in Iranians compared to Germans after controlling for training effects. Finger‐counting direction was not found to be decisive for this effect.

**Conclusion:**

At the end, number processing might be bilaterally represented in the IPS; however, our exploratory analyses emphasize the need for further investigation on the potential role of culture in the representations of numbers.

## Graphical abstract

This is the first cross‐cultural transcranial direct current stimulation (tDCS) study on functional lateralization of number processing. The cultural differences in reading direction did not influence functional lateralization of the intraparietal sulcus (IPS), pointing at a bilateral representation of number processing independent of culture.

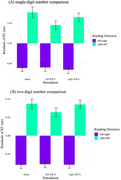

## 1 Introduction

Imagine the mind as a vast landscape, where numbers are not just random ideas but points on a mental number line, shaping our understanding of quantity. It was Francis Galton in the 19th century who first noticed that individuals do not perceive numbers as displaced entities hanging in their mind, but as spatially organized concepts that possess their own specific spot there (Galton 1880). The relations between number and space can either refer to extension (i.e., larger numbers are associated with a larger extension in space) or to directionality (i.e., larger numbers are located in a certain direction in space; Cipora et al. 2015, 2018; Patro et al. 2014). Cultural influences have been primarily, if not exclusively, demonstrated for directional space–number associations.

The hallmark effect of directional space–number associations is the Spatial–Numerical Association of Response Codes (SNARC) effect (Dehaene et al. 1993). Individuals tend to respond more quickly to small numbers with left‐sided responses and to large numbers with right‐sided responses—at least in Western cultures (Gevers et al. 2006; Wood et al. 2008). The SNARC effect has been repeatedly replicated in Western countries, and two different hypotheses are proposed to explain this effect: the innateness hypothesis and the acquisition hypothesis. The innateness hypothesis claims that directionality of space–number associations is shaped by biological factors including functional visuospatial asymmetries in early infancy (Nava et al. 2022), global (whole number)–local (decomposed fashion) processing (Pletzer et al. 2021), and asymmetry in brain frequency tuning (Felisatti et al. 2020). In contrast, the acquisition hypothesis argues for cultural factors shaping the directionality of space–number associations, including the effect of symbolic versus nonsymbolic notation of numbers (Cutini et al. 2014), directionality of reading system (Shaki et al. 2009), and finger‐counting habit (Tschentscher et al. 2012).

Beyond numbers, the functional lateralization of the brain—which contributes to differences in information processing and representations of functions (Willems et al. 2014)—is rooted in both structural asymmetries between the two brain hemispheres and experiences through enculturation and learning processes (Vallortigara et al. 1999). This paper investigates whether cultural factors, specifically reading direction, can influence the functional lateralization of number processing.

## 2 Innateness of Directionality

There is a hypothesis that the original underlying cause of directional preferences is innate: The innateness hypothesis proposes the idea of an underlying biological mechanism behind the mental number line. It argues, based on animal studies (e.g., Giurfa et al. 2022; McCrink and de Hevia 2018; Rugani et al. 2015) and developmental research on young infants (e.g., de Hevia et al. 2014; Macchi Cassia et al. 2016), that numbers are directionally ordered even in the absence of any cultural experience. For example, Rugani et al. (2015) trained 3 days‐old chickens to relative smaller and larger numerical content by walking them around panels with 5 or 20 items. Surprisingly, in test session they observed that when the number displayed on the panels was lower than the training number, chicks more likely walked to the left panel, while when it was higher than the training number, they walked to the right. Recently, they replicated their finding with rhesus macaques: monkeys performed better for left targets in arrays with two dots and for right targets in arrays with six or more dots, which is consistent with the left to right orientation of the mental number line (Drucker and Brannon 2014; Rugani et al. 2024). In a recent paper, brain regions subserving in newly hatched naïve chicks have been also identified (Lorenzi et al. 2024). In research complementary to animal studies, infant studies showed early spatial–numerical biases. de Hevia et al. (2014) observed that 7 months old Caucasian infants showed longer looking times when numerical displays were spatially displaced along a left to right orientation, discriminating between left to right, and other orders. To rule out early spatial–numerical learning, de Hevia et al. (2017) showed spatial–numerical associations even in 0–3 days old neonates. These observed biases in infancy and among animals are interpreted as untrained and spontaneous (for reviews see Rugani and de Hevia 2017 for spatial–numerical biases; Decarli et al. 2022 for generalizing the innateness hypothesis to numbers and [spatial] action).

Accordingly, the asymmetry in frequency tuning of the brain proposes a biological, non‐numerical mechanism for number–space associations based on asymmetric tuning of animal brains. Namely, different spatial frequencies in visual patterns of “few” versus “many” elements tend to engage right versus left brain hemispheres, respectively (Felisatti et al. 2020). This line of research suggests that number and physical space are inherently linked and that the directionality of the mental number line originates from certain biological mechanisms rather than learnt behaviors (Vallortigara 2018).

Nevertheless, it is important to note that, while culture‐specific spatial–numerical associations in preverbal infants cannot be attributed to habits like reading or finger counting, they may be linked to certain culture‐specific scanning routines, such as eye movements (Friedrich and Elias 2014; Göbel et al. 2018). Additionally, in an animal study, pretest experience affected the direction of space–number associations in 3‐day‐old chicks (Rugani et al. 2020).

## 3 Cultural Factors Shaping Directionality

According to the acquisition hypothesis, the interaction of space and number is influenced by embodied factors, which pertain to learning‐related aspects specific to each culture (Fischer 2024), and situated cognition factors, that consist of short time changes based on situational contexts. In this way, space–number associations are supposedly shaped throughout the course of development and through cultural experiences (Dehaene et al. 1993). For example, reading direction is correlated with where (i.e., on which side) participants choose to bisect, trisect, or quadrisect a line (Zivotofsky 2004).

Shaki et al. (2009) proposed that the cultural reading system serves as the most influential factor shaping the mental number line (Fischer and Shaki 2014; Göbel 2015; Shaki and Fischer 2008). Correspondingly, the SNARC effect was interpreted as reflecting a dependency between reading direction and the mental number line (Shaki et al. 2009).

According to this view, the SNARC effect should be observed in Western countries where the directionality of reading system is left to right; in Eastern countries with the opposite reading direction, the SNARC effect and the mental number line should be either reversed or absent. This assumption has been examined in cross‐cultural studies which found that Canadians with a left–right reading direction showed a regular SNARC effect, Palestinians with a right–left reading direction showed a reverse SNARC effect, and Israelis with right–left reading direction did not show any effect (Shaki et al. 2009; Zebian 2005). This suggests that the spatial orientation of the mental number line is the result of the directionality of the reading and writing system (Dehaene et al. 1993). However, the reverse SNARC effect (Shaki et al. 2009; Zebian 2005) could not be replicated. More recently, in an online SNARC experiment (Bulut et al. 2024), Iranians with a right–left reading direction also showed a regular SNARC effect—but the strength of the SNARC effect was modulated by reading direction; the strongest effect was found in Germany (left–right reading direction), then Turkey (recently left–right reading direction, while right–left reading direction one century ago), and the weakest was found in Iran (right–left reading direction). This suggests that reading direction as a cultural factor plays a role in the directionality of space–number associations, however, it is unclear whether a full reversal takes place. Interestingly, reading direction has been also reported to affect the perceptual asymmetries for aesthetic preference (Ishii et al. 2011), judgment of brightness (Nicholls et al. 1999), and perception of shape (Smith et al. 2015) and seems to affect a number of other directional activities (cultural directionality questionnaire in Bulut et al. 2024).

Additionally, reading direction may be just one of many cultural factors (including various early cultural directional experiences) which influence space–number associations (Bulut et al.; Nuerk et al. 2015; Patro et al. 2016). Since preliterate children have not yet acquired reading and writing skills, developmental research can be informative here. Studies have investigated counting direction habit, referring to the directional routine of pointing at objects and counting them, which can be evaluated through dot‐counting tasks (Shaki and Fischer 2024). A spatial left‐to‐right bias in counting direction has been observed in Western children even before reading acquisition (Opfer et al. 2018; Patro et al. 2015; Patro and Haman 2012; Shaki et al. 2012). Studies indicate that the SNARC effect is shaped by directional experiences starting in preschool and becomes more pronounced in elementary school. However, there is no clear evidence of a strong jump in the SNARC effect between preschoolers and elementary schoolers. This is challenging to assess because preschoolers are typically tested with nonsymbolic stimuli, while elementary schoolers use symbolic stimuli, complicating direct comparisons. These studies collectively underscore the developmental sensitivity of the SNARC effect suggesting that reading direction might not be the only factor influencing the mental number line.

Another cultural factor might be the directionality of finger‐counting habits. Fischer (2008) observed that two‐thirds of Scottish adults (left‐to‐right reading) prefer to start counting on the left hand (so‐called “left starters”). A cross‐cultural study confirmed that two‐thirds of Western adults map the numbers 1–5 onto the fingers of the left hand; this ratio between left and right starters was reversed for Iranian adults with right‐to‐left reading direction (Lindemann et al. 2011). Importantly, the starting hand in finger counting is related to the SNARC effect; cultures with a regular SNARC effect typically initiate finger counting with the left hand (Cipora et al. 2019; Fischer 2008). This association of finger counting and the SNARC effect however, did not replicate (Fischer et al. 2024).

Ultimately, finger‐counting direction could constitute one of the cultural factors that influence space–number associations (Nuerk et al. 2015). It follows that both reading direction and finger‐counting habits may be related to the directionality of the mental number line and as such influence the SNARC effect. However, like reading direction, finger counting has also received counterclaims. For instance, the cultural hypothesis, which emphasizes the embodied and situated nature of space–number associations, suggests that, although they can influence the mental number line, counting habit effects are not definitive due to their inherently variable nature. For example, a close relationship between reading direction and directional counting preferences was observed in languages with left‐to‐right and right‐to‐left reading directions, in both children and adults, with finger‐counting direction aligning with reading direction (Shaki et al. 2012). Extending these findings to the vertical dimension, the preferred counting direction in Cantonese adults was altered after reading a text presented in a different horizontal or vertical direction (Göbel 2015). In sum, there are clearly cultural factors influencing directional preferences like the mental number line.

Altogether, the debate on acquisition or inherency of space–number associations remains a controversial topic. Nevertheless, it is clear that, at least in addition to inherited space–number associations, cultural factors like reading direction and finger counting can shape the directionality of the mental number line (see McCrink and de Hevia 2018 for a model integrating innate biological factors and cultural factors).

## 4 Brain Lateralization for Space–Number Associations

The neural correlates of space–number associations provide further insights into the underlying mechanisms. For instance, patients with right‐hemisphere damage typically have a left‐hemifield neglect (i.e., paying attention mainly/only to the right side of their visual field; Marshall and Halligan 1990). In a line bisection task, these patients show a biased perceived midpoint to the right side of the center. This effect was also shown in numerical tasks: In number comparison, neglect patients showed an asymmetric distance effect, being slower for numbers smaller than (i.e., to the left of) the reference (Zorzi et al. 2012). In number bisection tasks, neglect patients consistently misjudged the midpoint on the mental number line, exhibiting a leftward bias for shorter intervals and an increasing rightward bias for larger intervals (Priftis et al. 2006). This suggests a role for hemispheric lateralization in space–number associations.

In the brain, space–number associations arise from common parietal circuits for attention to both external space and internal representations of numbers (Hubbard et al. 2005). This is in line with the Triple Code Model of numerical cognition, which suggests that numbers are represented bilaterally in the parietal cortex and, in particular, around the horizontal intraparietal sulci (Dehaene and Cohen 1997). This model has been extended by Klein et al. (2016), stating that the core of number processing around the bilateral intraparietal sulcus (IPS) is embedded in an interconnected frontoparietal network. Lateralization within this network was previously observed in a functional magnetic resonance imaging (fMRI) study: Despite any overt hand movements, the hemisphere contralateral to the hand used for counting small numbers was activated when small numbers were presented (Tschentscher et al. 2012).

Taking the past literature into account, the present study follows this argumentation:

- Cultures differ in their reading directions.
- This difference in reading direction is associated with differences in (finger) counting directions.
- According to Tschentscher et al. (2012), differences in (finger) counting directions are associated with a corresponding lateralization of the representations for small and large numbers.
- Consequently, modulating the different brain hemispheres should have differential effects for cultures with different reading directions.

Brain modulation is possible by applying transcranial direct current stimulation (tDCS) during number processing (for reviews, see Sarkar and Cohen Kadosh 2016; Schroeder et al. 2017a; Simonsmeier et al. 2018). tDCS delivers a weak electrical current through the scalp, which can either inhibit or excite cortical areas beneath the electrodes. This effect is achieved by modulating the likelihood of neurons generating action potentials and thereby altering brain activity in targeted brain regions. While bilateral IPS stimulation modulated number processing in one tDCS study (Klein et al. 2013), further tDCS studies support a functional lateralization for number processing within the IPS. First, Hauser et al. (2013) showed that anodal tDCS over the left IPS—but not cathodal tDCS or bilateral tDCS—increased accuracy in two‐digit number comparison (Hauser et al. 2013). Second, anodal tDCS over the right IPS impaired reaction time (RT) in single‐digit number comparison (Bahreini et al. 2023). Third, the SNARC effect was manipulated by cathodal tDCS over the left prefrontal cortex in single‐digit number comparison (Schroeder et al. 2017b). Altogether, evidence from tDCS points at a possible functional lateralization of number processing which can be altered by brain stimulation. Yet, all these findings were derived from samples within left‐to‐right reading direction cultures. Functional lateralization of number processing might be affected by culture, however.

The current study aims to evaluate the functional lateralization of the IPS in cultures with different directions in reading and finger‐counting systems by investigating number processing during single‐digit and two‐digit number comparison tasks. In a cross‐cultural tDCS study, we will compare samples from left‐to‐right (Germany) and right‐to‐left (Iran) reading systems and will further evaluate finger‐counting direction, which is estimated to be about two‐third left starters in Germany and two‐third right starters in Iran (Lindemann et al. 2011). To investigate the functional lateralization of the IPS in number processing, we used excitatory, anodal tDCS over the left versus right IPS during a number magnitude comparison task (cf. Bahreini et al. 2023; Hauser et al. 2013). As preregistered (Study 2, https://aspredicted.org/zj7r8.pdf), our hypothesis is that anodal tDCS over the left IPS, compared to sham, and over the right IPS, compared to sham, will differentiate RTs in number comparison tasks between left–right readers and right–left readers.

## 5 Method

### 5.1 Participants

An a priori power analysis was conducted to determine sample size using G*Power (version 3; Erdfelder et al. 1996). Sample size estimation for the effect size of Cohen's *d* = 0.2, with a power of 0.9 and *α* = 0.05 resulted in a minimum sample size of 106 participants.

The final sample consisted of 108 adults, including *N* = 54 German adults with left–right reading direction (18 males, age: *M* = 22.94 years, standard deviation (*SD*) = 3.84 years, Range = 18–34 years) and *N* = 54 Iranian adults with right–left reading direction (20 males, age: *M* = 25.52 years, *SD* = 3.23 years, Range = 18–50 years). This study is part of a larger project on functional lateralization of number processing; the German sample was taken from Bahreini et al. (2023). All participants were right handed as assessed by the Edinburgh Handedness Inventory (Oldfield 1971), with a cutoff ≥ 40 (*M* = 89.1, *SD* = 5.3). Furthermore, all participants were nonsmokers, native German speakers in the German sample and native Farsi speakers in the Iranian sample, with no history of neurological or psychiatric disorders. Additionally, tDCS exclusion criteria have been adopted.

Participants were recruited through circular emails, private contacts, and social media. As compensation, participants could receive either course credits or a monetary reimbursement. The study was conducted in accordance with the latest version of the Declaration of Helsinki; in Germany, it was approved by the local ethics committee for psychological research at the University of Tuebingen (Nuerk_2021_0902_235), and in Iran, it was approved by the ethics committee for human research at Iran University of Medical Sciences. Each participant gave their written informed consent for study participation and data sharing.

### 5.2 Number Comparison Task

**FIGURE 1:**
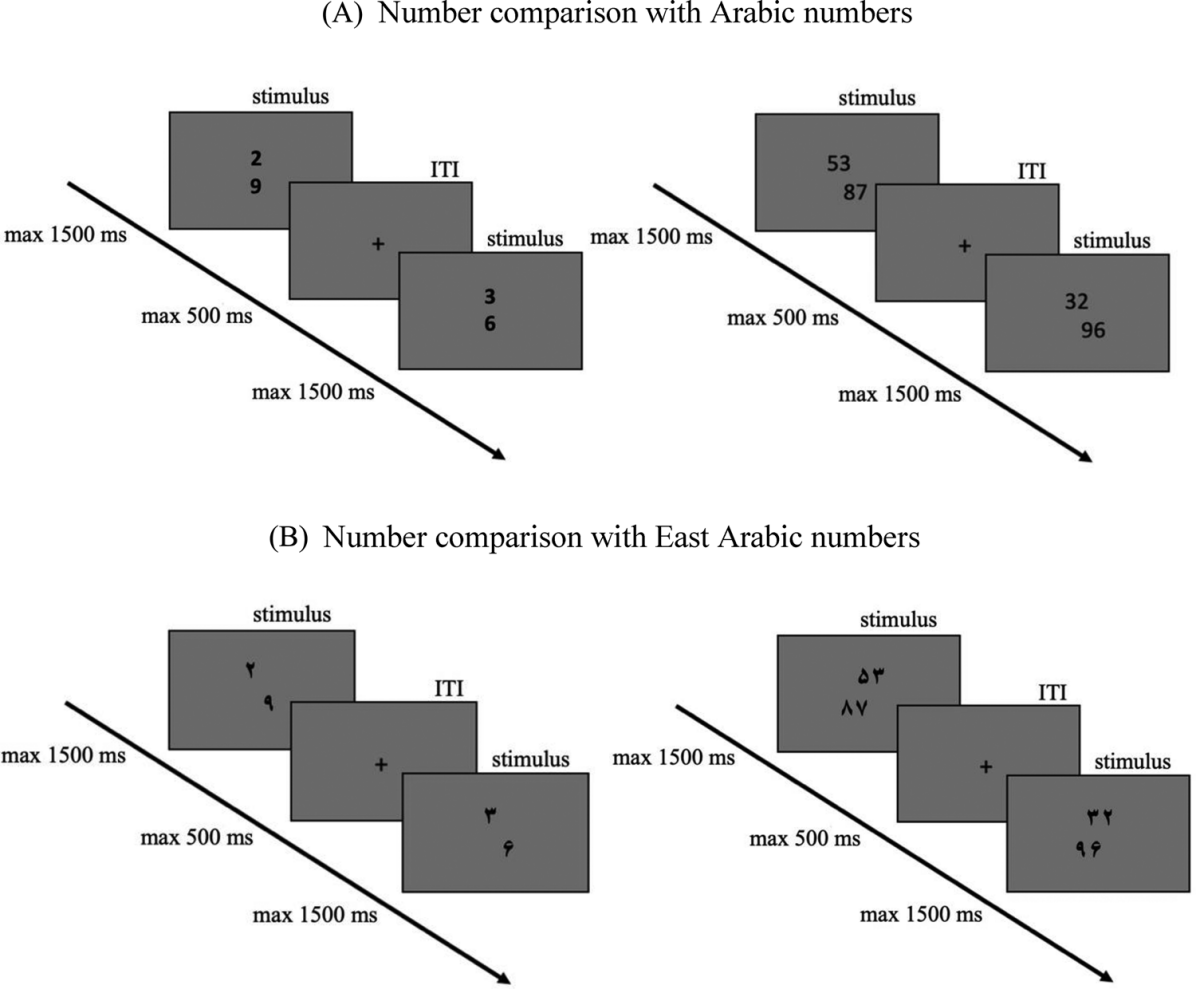
Single‐digit and two‐digit number comparison tasks: (A) Arabic numbers and (B) East Arabic numbers.

In a symbolic number magnitude comparison task (see Figure 1), participants were asked to judge as fast as possible which of two numbers is larger. Numbers were presented in Arabic notation for German participants and in East Arabic notation for Iranian participants. We chose East Arabic numbers for Iranians because they use this notation in cultural and language related contexts. The task was conducted in two versions: single‐digit and two‐digit number comparison (cf. Bahreini et al. 2023). Single‐digit number pairs consisted of 240 pairs of numbers between 1 and 9. For two‐digit number comparison, we used the stimulus set of Nuerk et al. (2001), consisting of 240 between‐decade pairs of numbers between 21 and 98. All number pairs consisted of four unique digits. Unit‐decade compatibility (i.e., unit and decade comparisons lead to the same decision, e.g., 56–41 with 5 > 4 and 6 > 1), problem size, and position of the larger number were counterbalanced across conditions. Task and stimulus set are openly shared (https://osf.io/g57av/).

The tasks were presented on a computer screen using PsychoPy (Peirce et al. 2019). The two numbers were displayed in Arial font, size 24, centered on a 21‐inch screen in a vertical arrangement; the numbers were horizontally jittered by one digit. The reason behind jittering was to prevent triggering the associations between magnitude and spatial location of the stimulus and the associated response key. The arrangement of response keys on the keyboard was up and down; half of the participants were instructed to respond using the upper key with their dominant hand and the lower key with their nondominant hand and vice versa. The response time limit was 1500 ms, and a fixation cross was presented for 500 ms between the trials.

### 5.3 Finger‐Counting Direction

Finger‐counting direction was assessed in two small tasks. In the first task, participants were presented with a drawing of both a right and left hand and asked to point at and count the fingers in the picture one by one. In the second task, participants were asked to count their own fingers from 1 to 10. To make sure that counting their own fingers was not influenced by the other task, they clapped their hands before starting finger counting. Participants were classified as left starters or right starters if all were counted in same direction.

To assess counting direction habit, participants were presented with four black dots in the center of a blank page and instructed to point with their finger at the dots and count them.

### 5.4 Transcranial Direct Current Stimulation

For the application of tDCS in the two countries, two devices of the same type were used: DC‐Stimulator MC (NeuroConn GmbH, Ilmenau, Germany). A current of 1 mA was applied on the head surface using rubber electrodes covered with saline‐soaked sponges. The active electrode (5 × 7 cm^2^) was placed over P3 or P4 of the international 10–20‐system and the reference electrode (10 × 10 cm^2^, current density of 0.01 mA/cm^2^) over the contralateral supraorbital region. Due to the large size of the reference electrode, this placement is expected to have minimum influence on the brain region underlying the reference electrode (Nitsche et al. 2008). The electrode arrangement of parietal cortex—contralateral supraorbital region was already successfully used in other tDCS studies (Artemenko et al. 2015; Hauser et al. 2013; Klein et al. 2010; Vines et al. 2006).

**FIGURE 2:**
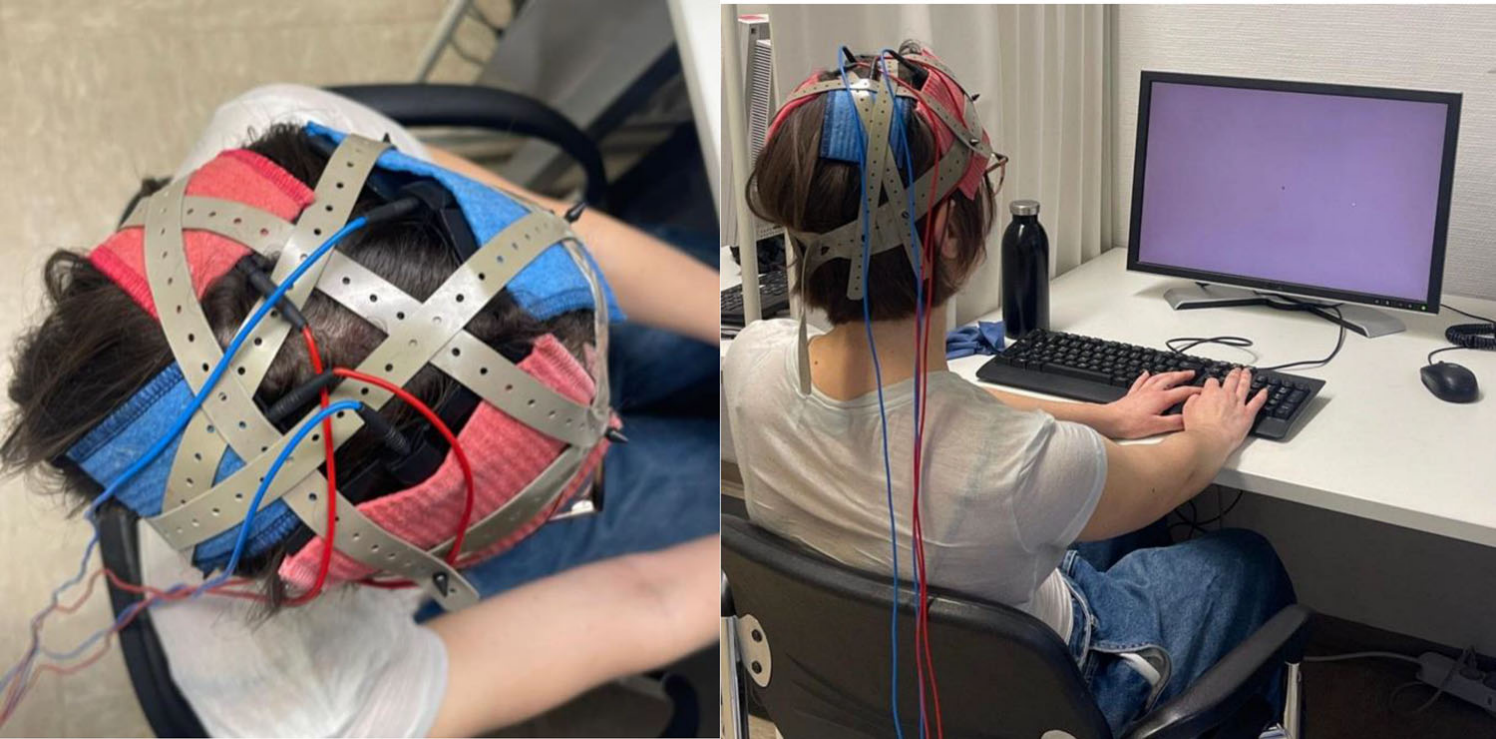
Transcranial direct current stimulation (tDCS) set up. Small red electrode is active one (in this picture placed over left IPS) and large red electrode is its return. Small blue sponge is active electrode for sham channel (placed over right IPS) and large blue electrode is its return. Returns are placed over supraorbital region. It should be noted that participant's consent for publishing these pictures is collected.

For active stimulation, the current was applied for 25 min and ramped up and down for 15 s. For left anodal tDCS, the target electrode was placed over P3, covering the left IPS according to the 10–20 system, with a reference electrode over the right supraorbital region; for right anodal tDCS, the target electrode was placed over P4, covering the right IPS, with a reference electrode over the left supraorbital region. In each stimulation condition, a bilateral electrode placement was used to blind participants with respect to the stimulation condition (see Figure 2). In a bilateral placement, one channel follows the experimental protocol while the other channel follows the sham protocol. For sham, the current was applied only for 30 s between the ramp up and down phases. This placebo condition is known to be indistinguishable from active stimulation for participants (Gandiga et al. 2006; Hauser et al. 2013). After completion of each session, participants were asked whether they could differentiate between real stimulation and sham. Only 11 out of 108 participants (10%) correctly guessed in all three sessions, so that blinding can be judged as successful in the current study. In the sham condition, based on our bilateral placement, one channel followed the sham protocol (with half of the participants receiving it on the left and the other half on the right), while the other channel delivered no current.

### 5.5 Procedures

In a single‐blind, within‐subject design, each participant received left anodal tDCS, right anodal tDCS, and sham across three separate sessions with a minimum intersession interval of 4 days (*M* = 5.94, *SD* = 1.53, Range = 4–10 days). The order of the stimulation conditions (P3, P4, and sham) was counterbalanced across participants. Each session followed the same procedure: 5 min after the onset of the respective tDCS protocol (25 min in total), participants conducted the two number comparison tasks. The order of the single‐digit and two‐digit number comparison tasks was counterbalanced across participants. For all participants, finger‐counting direction was assessed at the end of the sham session.

### 5.6 Data Preprocessing

Data analysis was conducted using R (Team 2021) and the ez (Lawrence 2016) and lmerTest (Kuznetsova et al. 2017) packages. Data were analyzed separately for single‐digit and two‐digit magnitude comparisons, in accordance with our preregistration (Study 2, https://aspredicted.org/6B3_59S). No participants had to be excluded due to technical problems, noncompletion of the study, or accuracy (proportion of correctly solved trials) lower than 75%. Overall accuracy for Germans was 97.10% (*M* = 0.48, *SD* = 0.11) in one‐digit number comparison, and 88.98% (*M* = 0.61, *SD* = 0.15) in two‐digit number comparison. For Iranians, overall accuracy was 99.07% (*M* = 0.58, *SD* = 0.17) in one‐digit number comparison, and 90.65% (*M* = 0.77, *SD* = 0.20) in two‐digit number comparison. For RT analysis, incorrect trials and RTs below 200 ms and above 1500 ms were excluded. Furthermore, and as preregistered, RTs above and below 3 *SD* of the participant's *M* were looked for to be excluded from the data in a repetitive trimming procedure separately for single‐digit and two‐digit number comparison. However, the data were so clean that this resulted in no (0%) exclusions.

### 5.7 Data Analysis

As preregistered, we conducted repeated‐measures analysis of variances (ANOVAs) and Bayesian ANOVAs for confirmatory analysis. For exploratory analysis, first we ran linear mixed‐effects models (LMMs) to control for the training effect. This study employed a mixed design, with each participant completing three sessions of the same number comparison tasks under different stimulation conditions; this led to training effects which might have masked possible stimulation effects and therefore should be controlled for (for the same effects and analysis, see (Bahreini et al. 2023). Next, an LMM was conducted to control for the potential effect of participants’ finger‐counting direction. This exploratory analysis aimed at investigating whether differential stimulation effects can be found based on finger‐counting direction as an additional cultural factor to reading direction. When both directions align and lateralization would differ between the cultures for small versus large single‐digit numbers (i.e., one hand vs. both hands), finger counting might play a role in functional lateralization of number processing. Last, we controlled for the possible effect of gender differences, conducting another LMM. Although no gender effect was reported by the studies presented in the current paper, gender has been reported as a factor in some brain stimulation researches on multidigit number processing (Knops et al. 2006; Pletzer et al. 2019). Therefore, we considered its potential contribution.

## 6 Results

### 6.1 Confirmatory Analyses for Reading Direction

Data and analysis script are openly shared (https://osf.io/g57av/). As preregistered, repeated‐measures ANOVAs and Bayesian ANOVAs with the within‐subject factor stimulation (left IPS vs. right IPS vs. sham), the between‐subject factor reading direction culture (left–right vs. right–left), and their interaction were conducted on RT separately for single‐digit and two‐digit number comparison. Paired *t*‐tests were further planned to compare stimulation conditions versus sham within each reading direction group.

**FIGURE 3:**
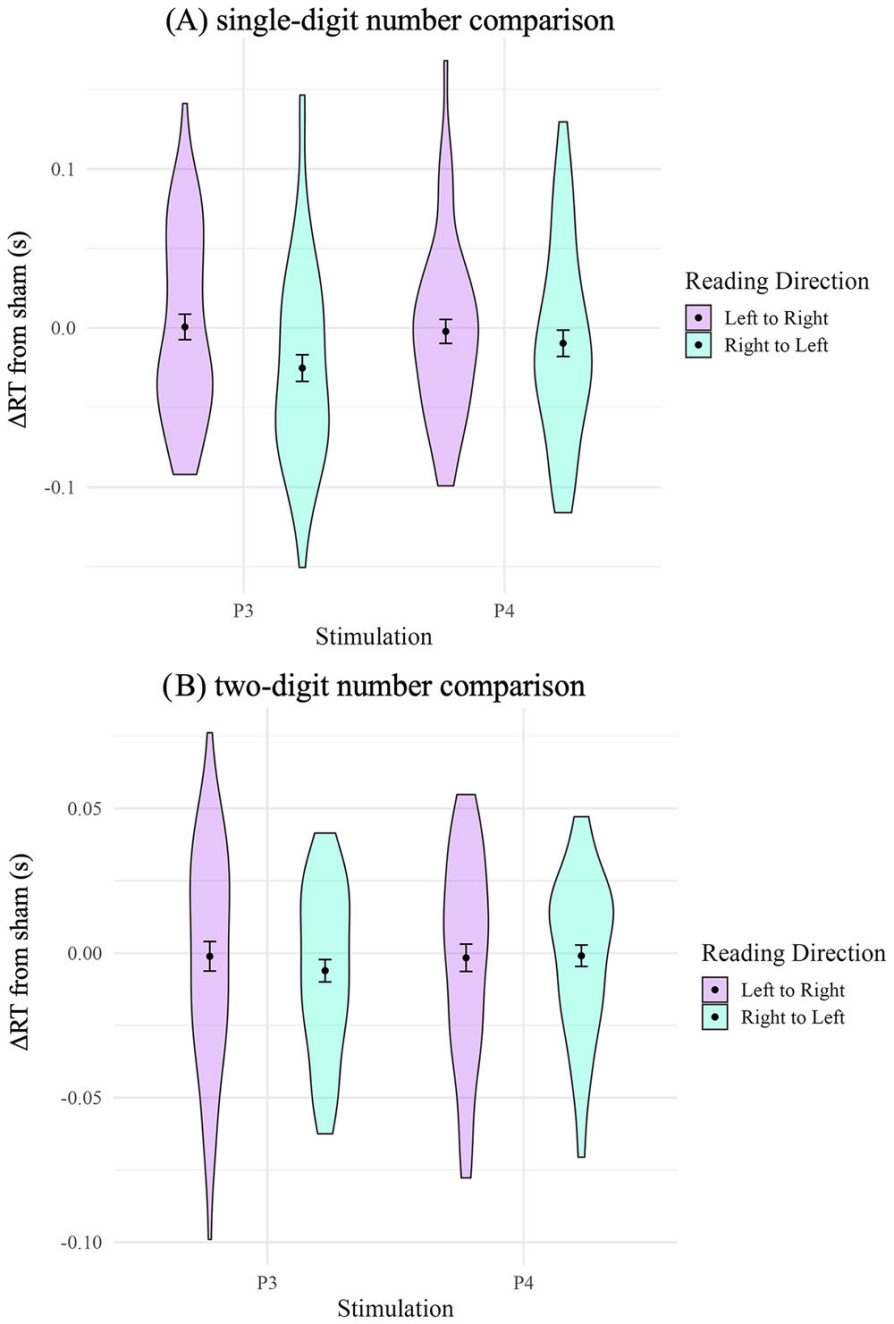
Confirmatory analyses show no transcranial direct current stimulation (tDCS) effects over the left and right intraparietal sulcus (IPS). Panel (A) single‐digit, Panel (B) two‐digit number comparison. The purple violins represent left‐to‐right reading direction (Germans), and mint green violins represent right‐to‐left reading direction (Iranians). Dots represent mean and error bars indicate standard error (*SE*).

The analysis of RT for single‐digit number comparison (overall *M* = 0.54, *SD* = 0.061 s) showed a significant main effect of reading direction culture (see Figure 3; *F*(1,106) = 132.112, *p* < .001, $\eta_{p}^{2}$ = 0.396), indicating that number comparison was significantly faster in Germans (left–right; *M* = 0.49, *SD* = 0.045 s) compared to Iranians (right–left; *M* = 0.59, *SD* = 0.068 s). There was no significant main effect of stimulation (*F*(2,212) = 1.494, *p* = 0.226, $\eta_{p}^{2}$ = 0.006), nor was there a significant interaction of reading direction culture and stimulation (*F*(2,212) = 1.761, *p* = .174, $\eta_{p}^{2}$ = 0.007). As mentioned in the preregistration, we complemented the result with a Bayesian analysis to better evaluate null effects (cf. Supplementary Material, Table S1). There was evidence for null effects of stimulation (BF_excl_ = 9.02) and of the interaction (BF_excl_ = 7.58). The *t*‐tests revealed no significant difference, with inconclusive data for tDCS over the left IPS versus sham (*t*(53) = 1.828, *p* = .070, BF_01_ = 1.51) and even a null effect for tDCS over the right IPS versus sham (*t*(53) = 0.699, *p* = .485, BF_01_ = 5.30) in Iranians. For Germans, null effects were found for both tDCS over the left IPS versus sham (*t*(53) = 0.0641, *p* = .949, BF_01_ = 6.61) and tDCS over the right IPS versus sham (*t*(53) = −0.367, *p* = .834, BF_01_ = 6.32).

The analysis of RT for two‐digit number comparison (overall *M* = 0.69, *SD* = 0.08 s) revealed a significant main effect of reading direction culture (see Figure 3; *F*(1,106) = 192.871, *p* < .001, $\eta_{p}^{2}$ = 0.483), indicating that number comparison was significantly faster in Germans (left–right; *M* = 0.617, *SD* = 0.073 s) compared to Iranians (right–left; *M* = 0.775, *SD* = 0.09 s). There was no significant main effect of stimulation (*F*(2,212) = 0.881, *p* = .415, $\eta_{p}^{2}$ = 0.004), nor was there a significant interaction of reading direction culture and stimulation (*F*(2,212) = 0.941, *p* = .391, $\eta_{p}^{2}$ = 0.004). We complemented the result with a Bayesian analysis (cf. Supplementary Material, Table S2), which provided evidence for null effects of stimulation (BF_excl_ = 17.46) and of the interaction (BF_excl_ = 25.93). The *t*‐tests revealed no significant difference and even null effects for both tDCS over the left IPS versus sham (*t*(53) = 1.283, *p* = .202, BF_01_ = 6.63) and tDCS over the right IPS versus sham (*t*(53) = 0.094, *p* = .925, BF_01_ = 6.48) in Iranians. For Germans, null effects were found for both tDCS over the left IPS versus sham (*t*(53) = −0.089, *p* = .928, BF_01_ = 3.27) and tDCS over the right IPS versus sham (*t*(53) = −0.144, *p* = .885, BF_01_ = 6.71).

### 6.2 Exploratory Analyses for Reading Direction by Controlling for Session

To control the training potential effect, we reanalyzed the data using LMM on RT including the fixed factors stimulation (left IPS, right IPS, sham), reading direction (left–right, right–left), and their interaction, with session (1, 2, 3) as a control factor and participants as a random factor. To obtain *p* values for the fixed effects we used the R package lmerTest, which calculates degrees of freedom using the Satterthwaite approximation. For model comparison, an automatic backward LMM selection procedure was applied which eliminates nonsignificant terms (*α* = 0.05 for both fixed and random effects) with the step function of lmerTest.

**FIGURE 4:**
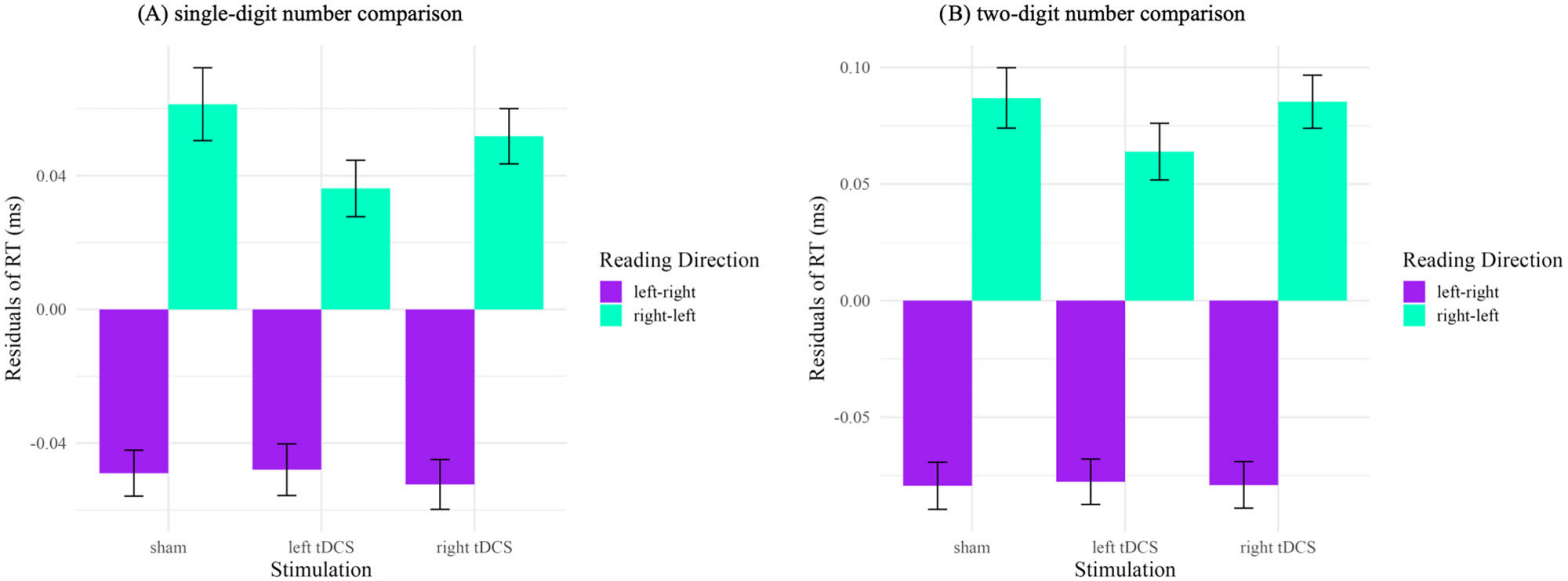
Cross‐cultural transcranial direct current stimulation (tDCS) effects in single‐ and two‐digit number comparison after controlling for session. Panel (A) single‐digit, Panel (B) two‐digit number comparison. Purple represents left‐to‐right reading direction, and mint green represents right‐to‐left reading direction. Residuals are standardized by the effect of session. Error bars indicate standard error (*SE*).

For single‐digit number comparison, the resulting reduced LMM was equal to the full model and included significant main effects for reading direction culture and session (see Table 1), indicating that Germans (left‐to‐right) were faster in number comparison than Iranians (right‐to‐left) and that participants were faster in sessions 2 and 3 compared to session 1. Additionally, interaction effects were found between stimulation and reading direction culture: Compared to sham, stimulation over the left IPS as well as right IPS facilitated the speed of number processing more for Iranians (right‐to‐left) than for Germans (left‐to‐right; see Figure 4).

**TABLE 1:** Results of the exploratory linear mixed‐effects model (LMM) analysis for reading direction.

Task	Effect	Estimate	Standard error (SE)	df	*t*‐value	*p*
Single‐digit	Stimulation (left vs. sham)	0.000	0.001	80,470	0.443	.658
	Stimulation (right vs. sham)	−0.003	0.003	80,510	−1.733	.083
	Reading direction (right–left vs. left–right)	0.110	0.008	111.8	12.567	< .001
	Session (2 vs. 1)	−0.018	0.001	72,130	−10.704	< 0.001
	Session (3 vs. 1)	−0.026	0.001	72,460	−15.146	< .001
	Stimulation × Reading direction (left vs. sham × right–left vs. left–right)	−0.025	0.002	80,470	−10.444	< .001
	Stimulation × Reading direction (right vs. sham × right–left vs. left–right)	−0.006	0.002	80,490	−2.575	< .001
Two‐digit	Stimulation (left vs. sham)	0.001	0.002	68,410	0.575	.565
	Stimulation (right vs. sham)	0.001	0.002	68,440	0.260	.795
	Reading direction (right–left vs. left–right)	0.165	0.001	112.1	14.572	< .001
	Session (2 vs. 1)	−0.001	0.002	62,210	−5.558	< .001
	Session (3 vs. 1)	−0.002	0.002	62,420	−9.727	< .001
	Stimulation × RrReading direction (left vs. sham × right–left vs. left–right)	−0.002	0.003	68,410	−7.359	< .001
	Stimulation × Reading direction (right vs. sham × right–left vs. left–right)	−0.001	0.003	68,430	−0.483	.629

*Note*: Estimates, *SE*, *t*‐ and *p*‐values of fixed effects in the reduced models, separately for single‐ and two‐digit number comparison.

For two‐digit number comparison, the resulting reduced LMM was equal to the full model and included significant main effects for reading direction culture and session (see Table 1), indicating that Germans (left‐to‐right) were faster in number comparison than Iranians (right‐to‐left) and that participants were faster in sessions 2 and 3 compared to session 1. Additionally, the interaction indicated that compared to sham, only stimulation over the left IPS decreased RT in Iranians compared to Germans (see Figure 4).

### 6.3 Exploratory Analyses for Finger‐Counting Direction

In another exploratory analysis, we further analyzed single‐digit number comparison and considered the congruity of reading direction and finger‐counting direction in each sample. Eighty‐five percent of Iranians were right starters (i.e., started counting with right hand), and 90% of Germans were left starters (i.e., started counting with left hand); thus, the sample included 15% left starters and 10% right starters in Iran and Germany, respectively. For further analysis, we excluded individuals whose finger‐counting direction was incongruent with their cultural reading system. Therefore, Germans with right‐to‐left finger counting (*n* = 5) and Iranians with left‐to‐right finger counting (*n* = 8) were excluded. Furthermore, we divided single‐digit numbers into two subsets: small, where both numbers in the pair are 1–5; and large, where both numbers in the pair are 6–9. We reanalyzed the data using the same LMM on RT including the factor subset and its interactions (see Table 2).

**TABLE 2:** Results of the exploratory linear mixed‐effects model (LMM) analysis for individuals with same reading and finger‐counting direction in single‐digit number comparison.

Effect	Estimate	Standard error (SE)	df	*t*‐value	*p*
Stimulation (left vs. sham)	0.001	0.004	12,710	0.215	.829
Stimulation (right vs. sham)	−0.001	0.004	12,710	−0.279	.780
Reading direction (right–left vs. left–right)	0.070	0.010	120	6.644	< .001
Session (2 vs. 1)	−0.020	0.004	9113	−4.890	< .001
Session (3 vs. 1)	−0.030	0.004	9784	−7.307	< .001
Stimulation × Rreading direction (left vs. sham × right–left vs. left–right)	−0.036	0.006	12,710	−1.357	< .001
Stimulation × Reading direction (right vs. sham × right–left vs. left–right)	−0.008	0.005	12,710	−2.569	0.175
Subset (small vs. large)	−0.080	0.003	12,710	−22.309	< .001
Subset × Reading direction (small vs. large × right–left vs. left–right)	0.030	0.005	12,710	6.552	< .001

*Note*: Estimates, *SE*, *t*‐ and *p*‐values of fixed effects in the reduced models.

We found significant effects for subset and its interaction with reading direction culture, indicating faster comparison of smaller subset as compared to larger subset—particularly in Germans as compared to Iranians. Furthermore, the interaction between stimulation and reading direction culture was significant. This indicates that stimulation over the left IPS versus sham facilitated single‐digit number comparison for Iranians with congruent reading and finger‐counting direction regardless of subset (see Figure S1). Importantly, this interaction was not significantly modulated by subset.

### 6.4 Exploring Gender Differences

Although none of the papers mentioned in the introduction reported a gender effect, and the largest SNARC study revealed no significant gender differences (Cipora et al. 2019), we explored the gender differences due to Bull et al. (2013) who reported sex differences in the SNARC effect. Therefore, we repeated the main analysis while including gender and no significant differences in the results were found (see Supplementary Material, Table S3).

## 7 Discussion

The purpose of this study was to investigate the potential influence of culture on functional lateralization of number processing. We hypothesized that the effect of cultural reading direction could be associated with hemispheric lateralization of number processing. Therefore, we expected to observe different stimulation effects after right and left IPS stimulation in Iranian and German samples due to their opposite reading direction. According to our confirmatory analysis, there was no interaction between culture and lateralized stimulation. While number comparison was significantly faster in Germans versus Iranians, the RT remained unaffected after stimulation of either the right or left IPS in both samples. Therefore, preregistered analyses did not show any indication of different hemispheric lateralization due to reading direction as a cultural factor. However, in exploratory analyses (LMM), after controlling for the training effect (cf. Bahreini et al. 2023), we found an interaction between stimulation of the right and left IPS with reading direction. Additionally, we found that the facilitatory effect of left IPS on Iranians remained when direction of finger‐counting and cultural reading direction were consistent.

### 7.1 Reading Direction Culture

Reading direction as a crucial cultural factor, contrary to our prediction, did not influence lateralization of number processing in the IPS. The idea that reading direction might be an influencing factor in hemispheric lateralization of number processing was inspired by the mental number line and SNARC studies that captured regular versus reverse SNARC effects in diverse reading direction cultures (Shaki et al. 2009; Zebian 2005). Our confirmatory results did not find any variation of stimulation effects by reading direction, which aligns with more recent SNARC studies which could not find a reverse effect in right‐to‐left reading cultures. For example, a regular SNARC effect was observed in Israelis (Zohar‐Shai et al. 2017) and in Iranians (Bulut et al. 2023) despite a right–left reading direction—but it is worth mentioning that the SNARC effect was nevertheless modulated by reading direction (Germany > Turkey > Iran). This suggests that reading direction as a cultural factor plays a role in the directionality of space–number associations; however, it is not clear whether a full reversal takes place—therefore, a different lateralization of space–number associations might be less probable.

Going beyond numerical cognition, directional spatial biases demonstrate a right hemispheric specialization for visuospatial processing with an attentional bias to the left side of visual space (for a review see Oleksiak et al. 2011). This hemispheric asymmetry might be limited to left‐to‐right readers: An Urdu (right‐to‐left) reader showed more activation of the left hemisphere in a case study (Rao et al. 2014). Furthermore, in a line bisection task, the midpoint of a horizontal line is typically placed slightly to the left of the actual center by left‐to‐right readers (for a meta‐analysis, see Jewell and McCourt 2000). One study found that this attentional bias could not be replicated in right‐to‐left readers (Chokron and Imbert 1993). However, subsequent studies revealed mixed findings. Some reported a reversal of the bias in right‐to‐left readers (Rinaldi et al. 2014; Smith et al. 2015), while others observed an absence of a group‐level bias (Friedrich and Elias 2014; Muayqil et al. 2021; Zivotofsky 2004). Additionally, some studies found similar biases in left‐to‐right and right‐to‐left readers, regardless of reading direction (Ishii et al. 2011; Nicholls et al. 1999). These conflicting findings highlight the influence of cultural reading habits on spatial attention but suggest variability in how reading direction shapes these biases across different tasks and contexts.

Although there is some evidence for an influence of reading direction on functional lateralization for spatial processing, it cannot be concluded that spatial processing varies by reading direction specifically for number processing. Even after controlling for training effects in the current exploratory analyses, only left‐sided stimulation facilitated number processing in right‐to‐left readers, whereas right‐sided stimulation did not facilitate number processing in left‐to‐right readers. Therefore, we conclude that the spatial representation of numbers is not influenced by reading direction.

Moreover, reading direction is not the only cultural factor that might influence number–space associations. Instead, it may be just one of many (early) cultural directional experiences (Nuerk et al. 2015; Patro et al. 2016) which influence space–number associations. Cultural preferences beyond reading direction may be more or less consistent with reading direction or even go into the opposite direction (Bulut et al. 2025)—this may eliminate or at least diminish brain lateralization of number processing imposed by reading direction, leading to the null results of our confirmatory analyses. In theory, the cultural hypothesis of hemispheric lateralization states that the information processing which creates an individual's cognitive style is shaped by cultural demands and will promote more use of either the right hemisphere or the left (Ornstein 1972). However, we could not find evidence for a cultural difference in lateralization in the current study.

Finally, number processing is considered to be bilaterally represented in the IPS according to the Triple Code Model (Dehaene et al. 2003) and its extensions (Klein et al. 2016). Although lateralization in the IPS has been identified for number processing, with the left IPS (Hauser et al. 2013; Sandrini et al. 2004), and for arithmetic, showing left dominance for addition/subtraction and right dominance for multiplication (Arsalidou and Taylor 2011), a cultural difference in functional lateralization was not observed in the current study. Instead, after controlling for training effects in our exploratory analysis, we found that stimulation of both the left and right IPS facilitate single‐digit number comparison for Iranians more than for Germans. In our previous study (cf. Bahreini et al. 2023), we observed that cumulative effects of training and task practice were substantial and thus seem to mask the effect of interest. As this part of the analysis was not preregistered, we refrain from making strong claims based on these findings and conclude only that lateralization does not differ, as both hemispheres exhibited similar effects. However, we strongly recommend always controlling for training effects in repeated‐measures designs in tDCS studies.

In sum, the expected differential functional lateralization of number processing based on reading direction culture was not found in the current study. Possible explanations might be that (1) the difference between reading directions in spatial–numerical associations is not as large as previously assumed, (2) lateralization of spatial processing in general might not generalize to numbers, (3) reading direction may not be the only cultural factor influencing lateralization, and (4) number processing might indeed be bilaterally represented in the brain.

### 7.2 Finger‐Counting Direction Culture

Reading direction is not the only cultural factor influencing number processing; the functional lateralization of number processing might also depend on earlier directional experiences in development, such as finger counting. The direction of finger counting and reading are mostly congruent in a culture. In our sample, 85% of Iranians were right starters (started counting with right hand) and 90% of Germans were left starters (started counting with left hand), which reflects the congruency of reading and finger‐counting directions. This is even slightly more than the previously found two‐thirds of congruent readers and counters of previous studies (Fischer 2008; Lindemann et al. 2011).

Therefore, we investigated functional lateralization of number processing for congruent reading and finger‐counting directions. The previously found exploratory result (after controlling for training effects) could be replicated, so that stimulation over the left IPS facilitated single‐digit number comparison for Iranians more than for Germans. However, the congruence between reading and finger‐counting direction did not modulate the stimulation effect for the small (1–5) versus large (6–9) subsets of single‐digit numbers in Iranian versus Germans. This suggests that the stimulation effect did not depend on the direction of finger counting, as the contralateral control of hands should have led to opposite lateralization effects for the different subsets (cf. Tschentscher et al. 2012). The reason might be that our stimulation target was the IPS, while previous research found finger‐based representations of numbers in the motor cortex (Tschentscher et al. 2012; see also Artemenko et al. 2022). As Tschentscher et al. (2012) reported lateralized effects only within the motor cortex, which is also part of the network underlying number processing (Klein et al. 2016), this might be a relevant stimulation site to be tested in future research. On the other hand, for investigating functional lateralization from a cultural overview, one important aspect to be considered is handedness of the participants (Laland 2008); however, this important factor is mostly ignored. For example, among the studies reported in this paper, only Zivotofsky (2004) had considered left handers (although no effect of handedness was found). Since our experiment only included right handers, the potential influence of handedness on finger‐counting direction could not be investigated. Ultimately, in the present study, the direction of finger counting within a reading direction culture did not significantly impact functional lateralization of number processing in the IPS.

### 7.3 Limitations

While this study offers valuable insights into the cultural effects on the neural underpinnings of number processing, it is important to acknowledge certain limitations. This is the first cross‐cultural tDCS study in this field, and it is not possible to control for all factors varying between cultures.

As mentioned above, one limitation of our experiment is the absence of left handers in each reading direction cultures. Unfortunately, reading direction culture and handedness could not be examined together in this study, even though they represent distinct research questions within a larger project on functional lateralization of number processing (https://aspredicted.org/zj7r8.pdf).

In this cross‐cultural study, the homogeneity of our German sample was notably higher, primarily due to recruitment via university email, whereas the lack thereof in our Iranian sample might have contributed to the slower RTs observed in Iranians compared to Germans, possibly because they were less familiar with experimental tasks. This higher familiarity with experimental tasks might explain why the German sample did not benefit from right‐sided stimulation, possibly due to a ceiling effect in performance. Also, we should emphasize that different number notation might have been a confounder.

## 8 Conclusion

Our cross‐cultural tDCS study investigated the role of culture in the neural representation of numbers, particularly emphasizing the influence of reading direction and finger‐counting habits on hemispheric lateralization in the IPS. Our confirmatory analysis showed no significant impact of reading direction culture on hemispheric lateralization of number processing and therefore provides support for a bilateral involvement of the IPS in number processing. However, when the effect of training was cancelled out in an exploratory analysis, we observed some cross‐cultural variation: Both left and right IPS stimulation facilitated number processing in Iranians more than in Germans. While we do not wish to make strong claims based on exploratory effects in the absence of confirmatory effects, we do suggest that these findings should be further investigated in future research. Finally, considering the consistency between reading and counting direction, we could not find evidence for a crucial role of finger counting in functional lateralization.

Moreover, our study finds several differences between Iranian and German samples. This suggests that not only behavioral, but also neuroscientific findings based on one (usually Western) sample should not be overgeneralized and seen as the standard cognitive processing in humans. Instead, we need to take cultural and interindividual differences more into account and move toward a more individualized approach, not only in behavioral but also neuroscientific studies.

## Author Contributions

**Narjes Bahreini**: conceptualization, data curation, formal analysis, visualization, writing–original draft, writing–review & editing. **Christina Artemenko**: conceptualization, validation, methodology, writing–review & editing. **Christian Plewnia**: conceptualization, methodology, writing–review & editing. **Reza Rostami**: resources, validation, writing–review & editing. **Hans‐Christoph Nuerk**: conceptualization, methodology, supervision, writing–review & editing.

### Peer Review

The peer review history for this article is available at https://publons.com/publon/10.1002/brb3.70353

## Supporting information



Supporting Information

## Data Availability

The datasets generated during the current study and the analysis scripts are available online (https://osf.io/g57av/).
