# Supplementary material for: Does the Brain Care Which Direction We Read? A Cross‐Cultural tDCS Study on Functional Lateralization of Number Processing
Source: Brain Behav. 2025 Mar 9;15(3):e70353. doi: 10.1002/brb3.70353 (PMC11891265; doi:10.1002/brb3.70353)
Supplement: Supplementary file 1 — Supporting Information [file BRB3-15-e70353-s001.pdf]

Supplementary Material

Does it matter for the brain in which direction we read and count? A cross-cultural tDCS study on functional lateralization of number processing

Narjes Bahreini, Christina Artemenko, Christian Plewnia, Reza Rostami & Hans-Christoph Nuerk

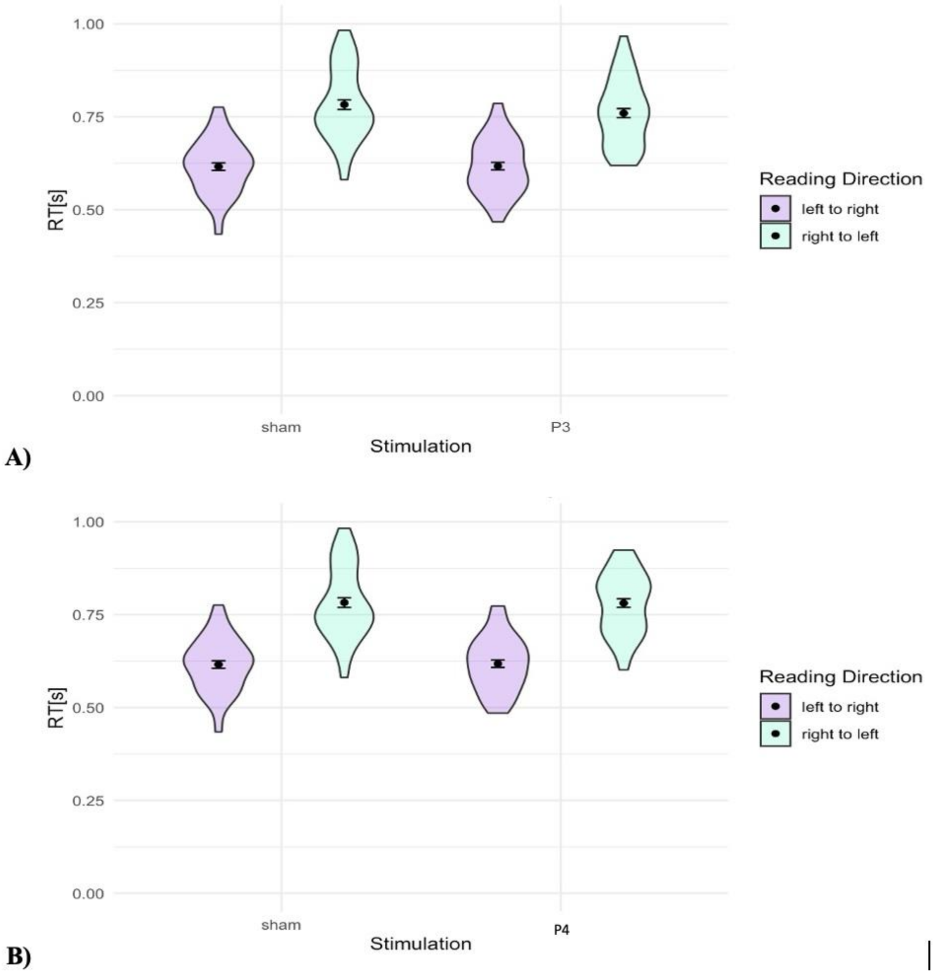

Figure S1 | Comparison of the numbers 1-5 during tDCS over the left and right IPS for different finger counting direction cultures. Participants with different finger counting direction are excluded. Error bars indicate standard error (*SE*).

Table S1 | Results of the Bayesian ANOVA for single-digit number comparison.

Model Comparison

**Models**	***P(M)***	***P(M|data)***	BF_M	BF_{01}	**error %**
Null model	0.200	< .001	< .001	1.000	
RD	0.200	0.858	24.075	< .001	1.699
stimulation + RD	0.200	0.111	0.497	< .001	1.249
stimulation + RD + stimulation × RD	0.200	0.032	0.132	< .001	1.784
stimulation	0.200	< .001	< .001	7.834	0.618

Analysis of Effects

**Effects**	***P(excl)***	***P(excl|data)***	BF_{excl}
stimulation	0.400	0.858	9.028
RD	0.400	< .001	< .001
stimulation × RD	0.800	0.968	7.581

Notes. RD: reading direction. BF_{excl} (Bayes Factor for exclusion) quantifies the evidence for excluding an effect against matched models with this effect. A value larger than 1 indicates evidence against the effect (null effect), while a value less than 1 indicates evidence for the effect.

Table S2 | Results of the Bayesian ANOVA for two-digit number comparison.

Model Comparison

**Models**	***P(M)***	***P(M|data)***	BF_M	BF_{01}	**error %**
Null model	0.200	< .001	< .001	1.000	
RD	0.200	0.921	46.559	< .001	1.015
stimulation + RD	0.200	0.070	0.299	< .001	1.861
stimulation + RD + stimulation × RD	0.200	0.010	0.039	< .001	1.919
stimulation	0.200	< .001	< .001	13.156	1.923

Analysis of Effects

**Effects**	***P(excl)***	***P(excl|data)***	BF_{excl}
stimulation	0.400	0.921	17.460
RD	0.400	< .001	< .001
stimulation × RD	0.800	0.990	25.936

Notes. RD: reading direction. BF_{excl} (Bayes Factor for exclusion) quantifies the evidence for excluding an effect against matched models with this effect. A value larger than 1 indicates evidence against the effect (null effect), while a value less than 1 indicates evidence for the effect.

Table S3 | Results of the exploratory LMM analysis for gender

Task	Effect	Estimate	*SE*	*df*	*t*-value	*p*
single-digit	stimulation (left vs. sham)	0.000	0.001	82050	0.533	.594
	stimulation (right vs. sham)	-0.001	0.001	82050	-1.069	.284
	reading direction (right-left vs. left-right)	0.110	0.008	110.5	12.44	< .001
	gender (male vs. female)	-0.003	0.009	105	-0.370	.711
	stimulation × reading direction (left vs. sham × right-left vs. left-right)	-0.025	0.002	82050	-10.32	< .001
	stimulation × reading direction (right vs. sham × right-left vs. left-right)	-0.007	0.002	82050	-2.909	.003
two-digit	stimulation (left vs. sham)	0.001	0.002	76,070	0.865	.387
	stimulation (right vs. sham)	0.002	0.002	76,070	1.068	.285
	reading direction (right-left vs. left-right)	0.164	0.011	110	14.302	< .001
	gender (male vs. female)	-0.006	0.012	105	-0.536	.593
	stimulation × reading direction (left vs. sham × right-left vs. left-right)	-0.022	0.003	76,070	-7.220	< .001
	stimulation × reading direction (right vs. sham × right-left vs. left-right)	-0.001	0.003	76,070	-0.540	.589

Notes. Estimates, *SE*, *t*- and *p*-values of fixed effects in the full models, separately for single- and two-digit number comparison.
